# A reproducible method for biochemical, histological and functional assessment of the effects of ischaemia–reperfusion syndrome in the lower limbs

**DOI:** 10.1038/s41598-021-98887-9

**Published:** 2021-09-29

**Authors:** Iñigo Cearra, Borja Herrero de la Parte, Diana Isabel Moreno-Franco, Ignacio García-Alonso

**Affiliations:** 1 Department of Traumatology and Orthopaedics, Osakidetza Basque Health Service, Basurto University Hospital, Avda. Montevideo, 18, 48013 Bilbao, Spain; 2 Department of Surgery and Radiology and Physical Medicine, Faculty of Medicine and Nursing, University of the Basque Country UPV/EHU, Barrio Sarriena s/n, 48940 Leioa, Spain; 3 Biocruces Bizkaia Health Research Institute, Plaza Cruces s/n, 48903 Barakaldo, Spain; 4 Department of Vascular Surgery and Angiology, Osakidetza Basque Health Service, Basurto University Hospital, Avda. Montevideo, 18, 48013 Bilbao, Spain

**Keywords:** Diseases, Preclinical research, Experimental models of disease

## Abstract

Current methodology described to mimic lower limb ischaemia–reperfusion injury (LL-IRI) does not accurately define the procedures and pressures exerted to induce and maintain ischaemia. In this piece of work, we propose a well-defined and detailed rat model that simulates the conditions established in clinical practice guidelines for tourniquet application and allows us to test treatments that aim to prevent/reduce LL-IRI. Eighty-six male WAG/RijHsd rats were subjected to hind limb IRI (LL-IRI), using a mechanical system applying a 1 kg tension to induce and maintain ischemia for 2 or 3 h, and assessed the damage caused by reperfusion at biochemical and muscular levels at different time points. At the biochemical level, both 2 and 3 h of ischemia induced changes (except for electrolyte levels); 3 h of ischemia induced greater changes in specific markers of muscular damage: creatine kinase (CK) and lactate dehydrogenase (LDH). At the histopathological level, 3 h of ischemia and 24 h of reperfusion was associated with an increase in hind limb girth, cross-sectional area, and weight and presence of neutrophils, as well as histological damage in more than 60% of muscle fibres. Our model allows to reliably reproduce the damage associated with the use of a pneumatic tourniquet. CK and LDH, as well as measures of tissue damage, allow to define and characterize the response to LL-IRI-related damage. A period of 3 h of ischemia followed by 3 h of reperfusion caused only local damage but showed greater sensitivity to detect differences in future studies on prophylactic treatments against LL-IRI.

## Introduction

Tourniquet-induced ischemia lasting for 1–2 h, or exceptional cases as long as 3 h, commonly achieved using a pneumatic cuff, is currently a routine process in extremity surgery, especially in orthopaedic surgery, allowing to obtain a bloodless surgical field, which facilitates surgical manoeuvres. However, its use entails a local ischemia/reperfusion injury (IRI). IRI has been widely described and studied in numerous sites and tissue types. Briefly, it is a systemic immune response, triggered by the release of proinflammatory mediators and reactive oxygen species during reperfusion of an organ or tissue that has undergone some period of ischaemia. This damage, due to immune activation during reperfusion, is actually greater than that caused by the ischemia itself^1,2^.

The clinical use of tourniquet cuffs has been associated with increased postoperative pain up to 2–6 weeks^3,4^. Further, greater swelling has been observed at 2^5^ and 6^6^weeks after surgery when tourniquet cuffs were used. Moreover, from a functional point of view, significantly poorer range of motion (ROM), and greater postoperative weakness of the quadriceps several weeks after the tourniquet was applied have been described^4–8^.

Several animal models of lower limb Ischaemia–reperfusion injury (LL-IRI) have been described to conduct experimental studies on these issues. However, there is poor consensus among them, both in terms of the animal model, the methodology employed, and the detailed description of the methodology, referring to them as "previously described by other authors". Literature on techniques for inducing LL-IRI includes invasive surgical procedures, involving muscle disinsertion and surgical excision or clamping of the femoral vessels^9,10^, to tourniquet models based on the application of a non-loosening nylon cable tie^11^, or rubber or latex bands^12–19^, used either in rats or mice. Both elastic tourniquets and high pressure levels applied with a uncontrolled tension tourniquet, such as rubber ring, increase both the frequency and severity of tourniquet-induced local injuries^20,21^. Among these animal models, only those models described by Bonheur^18^ and Drysch^19^ apply a model that is similar to that used in clinical practice, which uses the minimal limb occlusion pressure (MLOP), defined as the pressure in the tourniquet at which the distal arterial blood flow, as assessed by a Doppler probe held over a distal artery, is occluded.

An additional point of contention is whether ischaemia is performed on one (unilateral) or both (bilateral) limbs at the same time. Since, in the clinical settings, bilateral knee surgery might carry higher rates of blood transfusion, postoperative complications, increased incidence of 90-day mortality, venous or pulmonary thromboembolism and neurological complications^22–24^, it seems that bilateral models might further increase damages, masking the effects of the LL-IRI itself.

In preclinical research, imprecise description of some crucial aspects for the experiments, such as proper methodological description and specification of all the parameters, is a major obstacle to ensure its reproducibility^25^. Recently, in 2019, Sansa urged researchers to be more explicit and to publish ever more detailed experimental protocols, which allow others to repeat experiments. Moreover, he concluded that this "reproducibility crisis" could eventually undermine the general public's confidence in biomedical research, which might entail other unfortunate consequences^26^.

With that background, this piece of work details an experimental model in rats, based on several aspects of those already described by other authors, but closer to clinical practice, using a controlled tension system, as well as techniques and times similar to those employed in clinical practice. To quantify the LL-IRI, we have chosen those biochemical and histopathological parameters most related to the clinical use, among the many described in the scientific literature, and we have studied the best time of reperfusion to assess them. Additionally, we have also carried out physical studies to assess the degree of functional recovery after controlled ischaemia.

## Results

### Validation of the ischemia-inducing system

**Figure 1:**
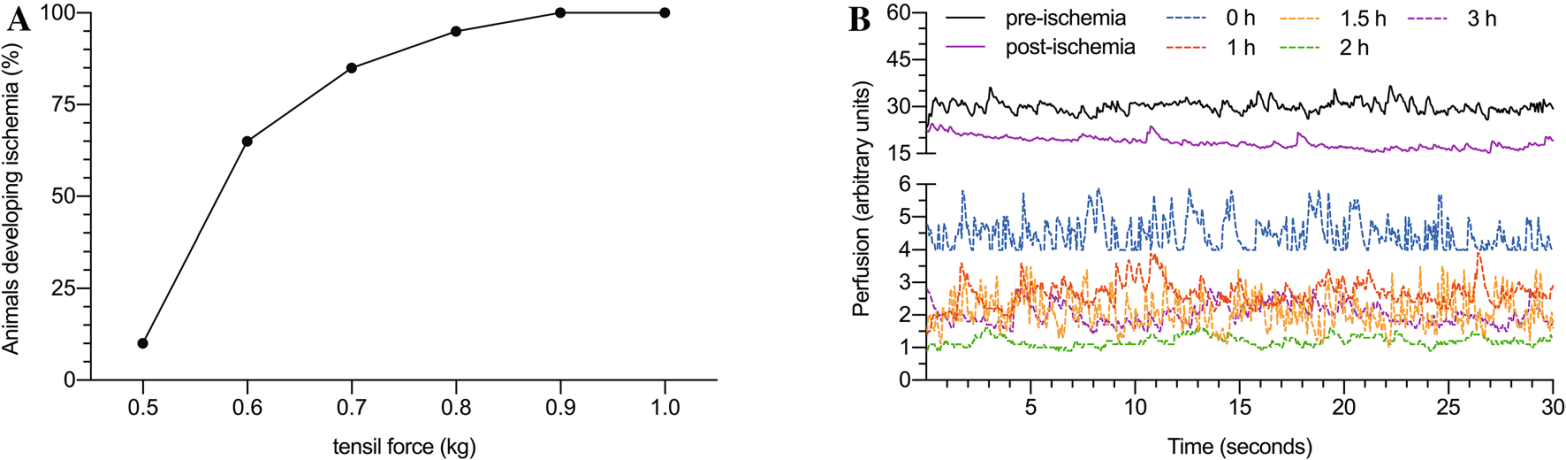
Percentage of animals developing ischemia under different tensile forces (**A**). Measurement of flow in the pedis artery territory in animals subjected to ischemia at 1 kg of tensile force (**B**).

All the procedures were well-tolerated, and no adverse events were reported. After applying an increasing tensile force ranging from 0.5 to 1 kg (with 0.1 kg increments), we found that it was necessary to apply 1 kg to achieve and maintain ischemia in 100% of the animals (Fig. 1A). These findings were confirmed both by the clinical signs described, but also by laser Doppler flow measurement (Fig. 1B).

### Biochemical assessment of LL-IRI

**Figure 2:**
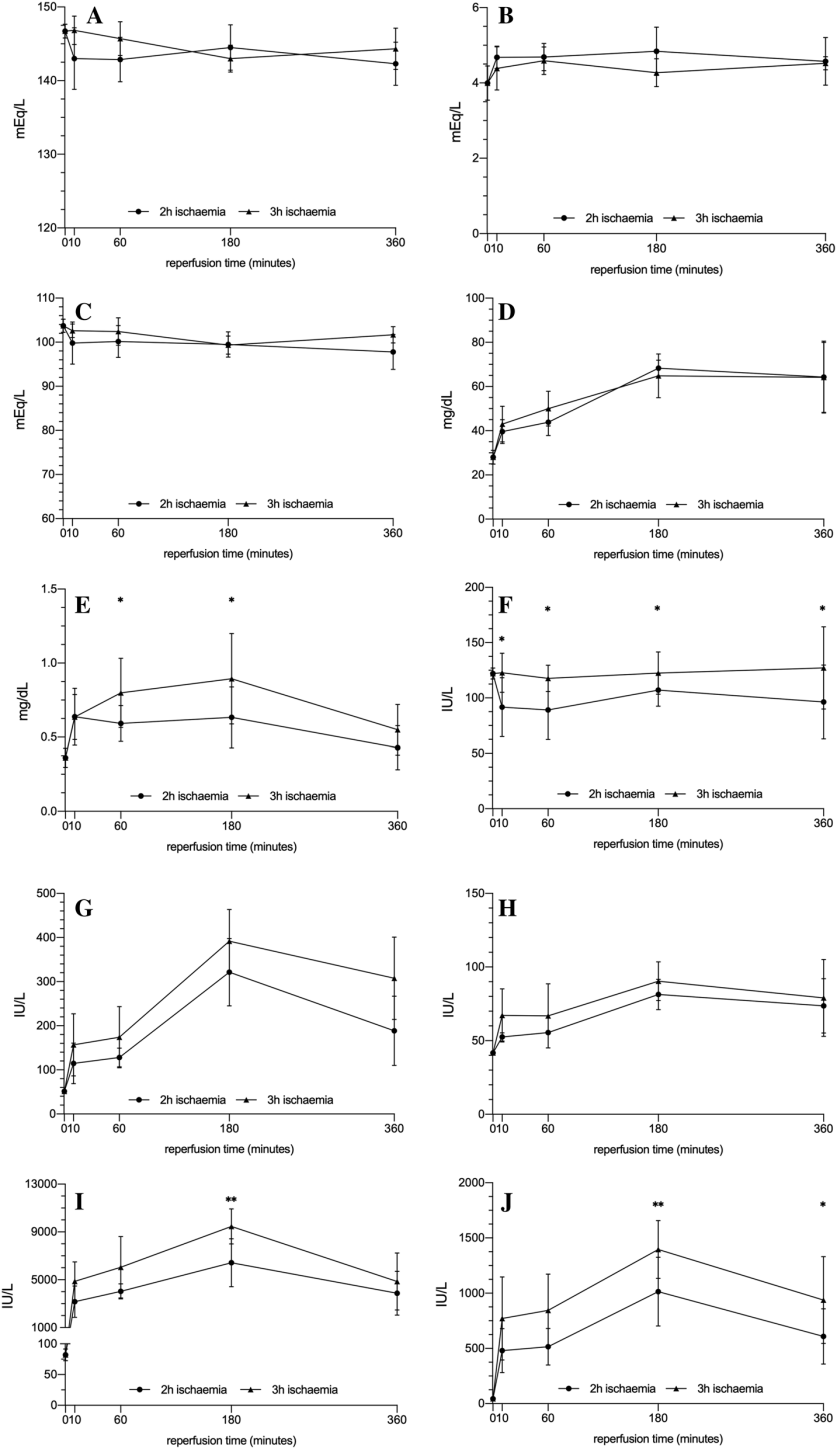
Measurements of serum electrolytes and enzymes of animals exposed to 2 h (circles, solid line) or 3 h (triangles, dashed lines) of ischemia and different periods of reperfusion. Na + (**A**), K + (**B**), Cl− (**C**), urea (**D**), creatinine (**E**), ALP (**F**), AST (**G**), ALT (**H**), CK (**I**) and LDH (**J**). Units are milliequivalents per litre (mEq/L) for Na^+^, K^+^ and Cl^−^, mg/dL for urea and creatinine, and international units per litre (U/L) for ALP, AST, ALT, CK, and LDH. The asterisks indicate statistically significant differences when comparing the two durations of ischemia (2 or 3 h) in each reperfusion period analysed; *(p < 0.05), **(p < 0.01). *ALP* alkaline phosphatase, *AST* aspartate aminotransferase, *ALT* alanine aminotransferase, *CK* creatine kinase, *LDH* lactate dehydrogenase.

The results concerning the biochemical parameters studied are presented in Table 1 and Fig. 2. In general, it can be seen that the greatest damage was seen in animals subjected to 3 h of ischemia, followed by 3 h of reperfusion.

**Table 1:** Results of biochemical analysis and levels of electrolytes in serum.

Group	Na^+^ (mEq/L)	K^+^ (mEq/L)	Cl^−^ (mEq/L)	Urea (mg/dL)	Creatinine (mg/dL)	ALP (U/L)	AST (U/L)	ALT (U/L)	CK (U/L)	LDH (U/L)
Control	146.7 ± 0.95	4 ± 0.45	103.7 ± 1.5	28 ± 3.2	0.36 ± 0.065	122 ± 5.06	51.43 ± 2.64	41.71 1.604	82.17 ± 9.41	44.83 ± 18.81
IR-1	143.0 ± 4.18 *n.s*	4.68 ± 0.29 *n.s*	99.8 ± 4.76 *n.s*	39.60 ± 5.36 *p* < *0.05*	0.648 ± 0.19 *p* < *0.05*	91.60 ± 26.6 *p* < *0.01*	114.8 ± 45.94 *n.s*	52.50 ± 2.77 *n.s*	3168 ± 1314 *p* < *0.01*	480 ± 199 *p* < *0.01*
IR-2	142.9 ± 3.02 *n.s*	4.68 ± 0.36 *n.s*	100.1 ± 3.62 *n.s*	43.86 ± 6.01 *p* < *0.01*	0.539 ± 0.12 *p* < *0.05*	89.17 ± 26.7 *p* < *0.0001*	128.0 ± 21.44 *n.s*	55.50 ± 0.55 *n.s*	4025 ± 629.7 *p* < *0.001*	515 ± 166 *p* < *0.01*
IR-3	144.5 ± 3.08 *n.s*	4.83 ± 0.64 *n.s*	99.5 ± 2.88 *n.s*	68.33 ± 3.61 *p* < *0.001*	0.633 ± 0.20 *p* < *0.05*	107.0 ± 14.68 *n.s*	321.5 ± 76.38 *p* < *0.001*	81.33 ± 10.26 *p* < *0.0001*	6429 ± 2007 *p* < *0.0001*	1015 ± 310.8 *p* < *0.0001*
IR-4	142.3 ± 2.93 *n.s*	4.5 ± 0.63 *n.s*	97.8 ± 3.96 *n.s*	64.29 ± 16.3 *p* < *0.001*	0.428 ± 0.14 *n.s*	96.39 ± 36.44 *n.s*	188.67 ± 104.0 *p* < *0.001*	73.67 ± 18.50 *p* < *0.0001*	3872 ± 1834 *p* < *0.001*	609 ± 250.8 *p* < *0.001*
IR-5	147.2 ± 1.92 *n.s*	4.38 ± 0.57 *n.s*	102.6 ± 1.52 *n.s*	43.0 ± 8.09 *p* < *0.05*	0.64 ± 0.15 *n.s*	122.8 ± 17.77 *n.s*	156.8 ± 70.33 *p* < *0.05*	67.20 ± 18.10 *n.s*	4870 ± 1623 *p* < *0.001*	770.4 ± 376.4 *p* < *0.01*
IR-6	145.7 ± 2.29 *n.s*	4.59 ± 0.36 *n.s*	102.4 ± 3.1 *n.s*	50.0 ± 7.81 *p* < *0.001*	0.80 ± 0.23 *p* < *0.01*	117.66 ± 11.91 *n.s*	174.1 ± 69.43 *p* < *0.01*	66.86 ± 21.73 *n.s*	6042 ± 2570 *p* < *0.0001*	844.0 ± 328.2 *p* < *0.01*
IR-7	142.7 ± 1.75 *n.s*	4.27 ± 0.37 *n.s*	99.3 ± 2.06 *n.s*	64.8 ± 9.91 *p* < *0.001*	0.88 ± 0.31 *p* < *0.001*	122.5 ± 19.17 *n.s*	391.8 ± 72.0 *p* < *0.001*	90.33 ± 13.19 *p* < *0.001*	9459 ± 1467 *p* < *0.0001*	1395 ± 262.4 *p* < *0.0001*
IR-8	144.3 ± 2.8 *n.s*	4.52 ± 0.17 *n.s*	101.7 ± 1.86 *n.s*	64.2 ± 15.8 *p* < *0.001*	0.55 ± 0.17 *n.s*	127.16 ± 37.18 *n.s*	307.5 ± 93.35 *p* < *0.001*	79.00 ± 26.1 *p* < *0.01*	4858 ± 2392 *p* < *0.001*	937.5 ± 393.3 *p* < *0.001*

Data are presented as the mean, the corresponding standard deviation (SD) and the p value from comparison with the reference value of the control group (first line), considering p values < 0.05 to be significant. *ALP* alkaline phosphatase, *AST* aspartate aminotransferase, *ALT* alanine aminotransferase, *CK* creatine kinase, *LDH* lactate dehydrogenase.

Regardless of the duration of ischemia and subsequent reperfusion, the levels of electrolytes analysed (Na^+^, K^+^ and Cl^−^) did not vary significantly from reference values (Fig. 2A–C; Table 1). The ischemia/reperfusion process led to an increase in urea and creatinine levels, the indicators of kidney function (Table 1). In the case of urea, a progressive increase was observed throughout the reperfusion process, with a peak after 3 h, with both durations of ischemia (2 h: 65 ± 9.9 mg/dL; 3 h: 68 ± 3.6 mg/dL); reaching 2.3-fold higher than levels in controls (28 ± 3.2 mg/dL; p < 0.001). One hour more of ischemia did not produce significant a change in urea levels (Fig. 2D). In contrast, serum creatinine levels were higher in the animals exposed to a longer duration of ischemia (Fig. 2E; p < 0.05). Specifically, with 2 h of ischemia, the peak was reached after 10 min of reperfusion (0.648 ± 0.19 vs 0.36 ± 0.065 mg/dL in controls; p < 0.05), the level staying high until 3 h, whereas after 3 h of ischemia, creatinine levels continued increasing until a peak of 0.88 ± 0.31 mg/dL at 3 h.

In our experiments, the ischemia/reperfusion was not associated with increases in ALP activity (Fig. 2F). In fact, there were no significant changes in the levels of this enzyme in the animals exposed to 3 h of ischemia, while in those exposed to 2 h of ischemia, levels decreased significantly during the reperfusion period.

Regarding other ubiquitous enzymes, levels of AST and ALT (Fig. 2G,H, Table 1) progressively increased until 3 h; at this point, they started to decrease but did not reach normal levels by 6 h. Though the pattern was similar in the two enzymes, the peak was more dramatic in the case of AST (391.8 ± 72 IU/L) than ALT (90.33 ± 13.19 IU/L).

Levels of CK, a very specific serum marker of muscle damage, followed a very similar pattern to those of AST, with a threefold increase in the first 10 min of reperfusion and a peak at 3 h (Fig. 2I). At all times, the serum levels of this enzyme were significantly higher in animals exposed to 3 h of ischemia rather than 2 h (3-h exposure: 9459 ± 1467 IU/L vs 2-h exposure: 6429 ± 2007 IU/L, p < 0.01). As in the case of AST, CK levels did not reach normal values after 6 h of reperfusion.

Lastly, LDH levels (Fig. 2J, Table 1) also behaved similarly, with levels increasing markedly after just 10 min of reperfusion, reaching levels significantly higher than in controls at this point (44.83 ± 18.81 IU/L), and peaking at 3 h (1395 ± 262.4 IU/L; p < 0.01). In animals subjected to 3 h of ischemia, serum levels of LDH were higher throughout follow-up and did not return to normal after 6 h of reperfusion.

### Anatomopathological evaluation of LL-IRI

**Figure 3:**
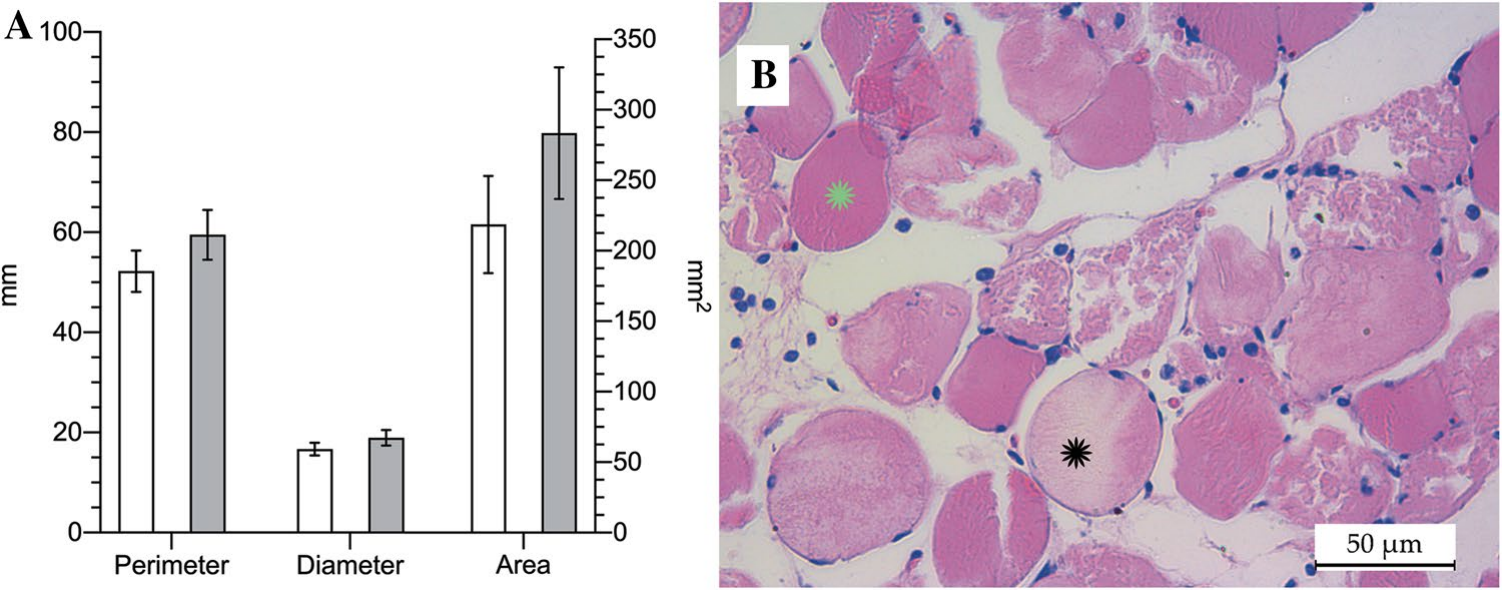
Upper-thigh girth and cross-sectional area of the legs exposed to 3 h of ischemia and 24 h of reperfusion (grey) and the other legs, used as healthy controls (white). Girth and cross-sectional area are expressed in millimetres (mm, left axis) and millimetres squared (mm^2^, right axis), respectively (**A**). Representative histological section of a limb subjected to ischemia–reperfusion (the green asterisk indicates an undamaged muscle fibber and the black asterisk shows a muscle fibber damaged by ischemia–reperfusion. The extensive polymorphonuclear cell infiltrate (stained in blue) and oedema between the muscle fibbers (white space) are also seen) (**B**).

Twenty-four hours after removal of the tourniquet, the mean upper-thigh girth of the leg exposed to ischemia/reperfusion was 59.5 ± 4.96 mm, 13% larger than that of the other leg (control, 52.25 ± 4.13 mm; p < 0.0001). This increase in girth translates to an estimated 30% increase in the cross-sectional area: 283.5 ± 46.71 mm^2^ vs 218.6 ± 34.50 mm^2^ in the control leg (p < 0.0001) (Fig. 3A). Regarding the weight of the gastrocnemius muscle, we observed a nearly 10% increase, with a mean muscle weight of 1.98 ± 0.19 g for the legs exposed to the ischemia vs 1.81 ± 0.14 g for the control legs (p < 0.0001).

In the microscopic assessment (Fig. 3B), in animals exposed to 3 h of ischemia and 24 h of reperfusion, we observed alterations compatible with IRI-related damage in 65.53 ± 6.86% of fibres in the gastrocnemius muscle. The mean PMN count was 55.28 ± 9.73 cells/field, corresponding to approximately 4.52 × 10^4^ cells/mm^2^.

### Rotarod test

**Figure 4:**
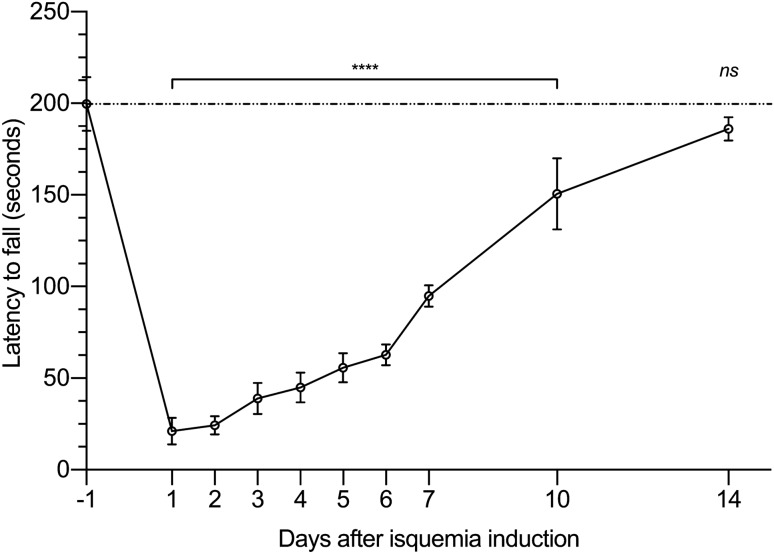
Rotarod test. The data shows the mean and standard deviation in the fall time (expressed in seconds) of the animals subjected to 3 h of ischaemia period; ns (p > 0.05), ****(p < 0.0001).

Figure 4 shows the results obtained with the rotarod test. Because of the effects of the anaesthesia used during the ischaemia period, the test could not be performed on the day of the ischaemia (day 0). One day after ischaemia (day + 1, 21.17 ± 7.25 s), a marked decrease in fall latency time was observed, compared to the control, obtained one day before ischaemia (day-1, 199.58 ± 14.75 s; p < 0.0001). Despite a progressive increase in the latency to fall time up to the 10th day (150.5 ± 19.54 s), highly significant differences could still be observed, compared to the pre-ischemia benchmark time. Nevertheless, these differences were no longer observed in the 14th day (186 ± 6.39 s), when the latency to fall time is already similar to the time recorded before the ischaemia.

## Discussion

Although numerous LL-IRI-related experimental studies have been conducted, the experimental models described in the literature are notably different, there being no consensus on a standard model. The differences are evident in various characteristics of the models including the method of inducing and maintaining the ischemia and the parameters used to assess damage as well as when it is assessed. This diversity makes it very difficult to evaluate the validity and efficacy of treatments tested, and warrants research to identify a model that is reproducible and reliable, as well as easily transferable to clinical practice.

An important question is the choice of the experimental animal. Several considerations have led us to decide in favour of using rats for the research. First, they have more similarities to humans, both in the anatomy of the lower limb muscles having the same muscle groups^27,28^, and in the tolerance of tissues to ischemia and their response at biochemical and histopathological levels^29–32^. In fact, this is the most widely used animal model in the study of LL-IRI^9–16^. It is also a species that is easy to handle and has gentle behaviour and suitable size, facilitating preoperative management and husbandry. Finally, our research group has proven experience in the handling of this species for the study of IRI in other body systems^33–35^.

A second important question for establishing a good IRI model is which type of device or procedure to use to induce and maintain the ischemia. Published studies have used very simple devices, based on a ring or tube, that do not allow measurement of the pressure being applied to maintain the ischemia, and in most cases, apply an excess pressure leading to muscle damage resulting from crushing (crush syndrome), distorting the assessment of damage due to IRI and making the model unlike clinical practice^9–16,36^. For this reason, one of the goals of this study was to develop and optimise a mechanical system that imitates the gold standard used in clinical practice, namely, a pneumatic cuff, with which we can induce ischemia using as little pressure possible, and thereby, minimise the damage resulting from the compression.

It is difficult, however, to place a pneumatic cuff on a rat´s leg. First of all, due to the small size of the cuff that would be needed^37,38^. Further, the truncated cone shape of the leg makes it very difficult to avoid it moving once inflated. To avoid these problems, finally we opted for a system of tension cables, similar in concept to the controlled tension tourniquet described by Bonheur in a murine model^18^. In mice, 0.2 kg was sufficient to maintain the ischemia; in the case of rats, we have found it necessary to use 1 kg to achieve a suitable level of ischemia. In a previous study of retinal ischemia–reperfusion^39^ conducted in WAG/RijHsd rats of similar sex, age, and weight to those used in the present study, we determined a mean arterial pressure (MAP) of 126 ± 4.6 mm Hg; in different mouse strains, the MAP ranged from 108 to 114 mm Hg ^40^. This difference in MAP does not seem to be enough reason to justify a fivefold increase in tensile strength necessary to induce the onset of ischemia in the animals. The most plausible explanation for this difference appears to be simply the difference in size between the two species, but might also be related to the thickness of the cable used. We also believe that the support arms, one to keep the cable stable and one to apply gentle traction to the end of the leg, are useful extra features of our design that allow us to ensure that there are no undesired movements or loss of ischemia.

A third key question is which parameters to use to assess the damage. Among those reported in the literature, we have opted for those which are closest to the parameters used in clinical practice.

A considerable number of clinical studies, mainly focused on assessing the use of anaesthetic drugs to control the damage due to reperfusion immediately after removing a tourniquet, consider the measurement of ischemia modified albumin (IMA), isoprostanes-isofurans and malondialdehyde (MDA)^41–45^*.* Nonetheless, we believe that the use of these parameters, as well as being expensive, is not easily transferrable to clinical practice. In contrast, both CK and LDH have been widely studied in experimental studies^11,14,46,47^, are good indicators of rhabdomyolysis and are used in clinical practice^48–50^. In line with these studies, we observed a sudden increase in the levels of both of these enzymes, from the onset of the reperfusion process. Further, their pattern is highly compatible with what Murata et al. described in their crush syndrome model^14^*.* In our case, the levels of these two enzymes peaked after 3 h of reperfusion, while other authors^11^ reported a peak after 4 h. To our mind, it is very interesting that, after a sudden increase immediately after the start of the reperfusion (observed at 10 min) and the levels remaining more or less stable until 60 min, there is a second increase by 3 h. We could speculate that the initial increase after just 10 min seems to be too early to be due to reperfusion. In fact in vitro studies show that skeletal and cardiac muscle fibbers submitted to anoxia release significant amount of LDH and CK^51,52^.

The rest of the biochemical markers analysed (electrolytes, markers of kidney function and other nonspecific enzymes) have been studied by other authors in different models of lower limb ischemia^2,11,14^. Findings with our model are fairly similar to their results, in terms of a lack of changes in electrolyte and ALP levels, and slight increases in AST and ALT levels after long periods of reperfusion.

Finally, the markers of kidney damage (urea and creatinine), widely used in IR models, show different patterns. The increase in urea levels continues beyond the period of reperfusion studied, while creatinine increases early on, its levels returning to normal after 6 h.

We should also note that some studies in the literature mention the use of indicators of acid–base balance such as lactate, pH or the anion GAP. These tend to be considered, however, for assessing longer-lasting ischemia and higher compressive forces with potential systemic complications^14,53,54^, and we have not measured them in our model, which only causes local damage.

Although ischemia does not tend to be induced for longer than 2 h in clinical practice, our observations indicate that with ischemia lasting 3 h, the damage remains local and reversible, as expected^30,32,55^, although it is obviously increased. For this reason, we believe that 3 h is an ideal duration of ischemia for testing experimental treatments, providing the opportunity to observe any potential protective effects more clearly. In the case of biochemical analysis, based on our own experience and findings of other studies^11,14^, we believe that measurements should be made after 3 h of reperfusion.

Assessment of histopathological damage is the most controversial and a matter of debate in the literature. While it would be possible to measure substances such as myeloperoxidase, MDA or glutathione by spectrophotometry, this approach is not routinely used in hospitals, and hence, results would be difficult to translate to clinical practice and to interpret, and additionally, the measurements are technically complicated and expensive^12,14,15^. For this reason, we have again opted for parameters that are more commonly used in clinical practice, in particular, direct assessment of tissue swelling by measuring leg girth, which is easy to perform in patients.

In our model, the swelling present after 24 h of reperfusion increased the upper-thigh cross-sectional area by 30%. This figure is similar to those from a rabbit experimental model^56^, in which there was a 38% in the cross-sectional area after 4 h of ischemia and 24 h of reperfusion. Regarding the weight of the gastrocnemius muscle, we observed a 10% increase, while Bonheur^18^ reported a 22% increase, after 3 h of ischemia and 24 h of reperfusion. The difference may be due to Bonheur having used mice, which are a priori more fragile and sensitive.

Other parameters assessed in the literature include histological damage and leukocyte infiltration assessed by microscopy^14,17,18,36,56^. Our results quantifying microscopic damage, expressed as the percentage of damaged fibres per field, in accordance with the method described by McCormack^36^, indicate that two-thirds (66%) of fibres were damaged. This is in line with figures reported by other authors, who have found damage in up to 25% of fibres after 90 min^17^ and 80% after 4 h^56^ of ischemia. Finally, the PMN count obtained was 4.52 × 10^4^ cell/mm^2^, consistent with the findings of Deune et al. in a rabbit model with ischemia lasting for 4 h^56^.

Finally, it is of outmost importance that experimental models translate pathological and biochemical damage into functional impairment or recovery, as this late aspect is what really matter in the clinical setting. In fact, several clinic studies in this field include postoperative mobility indexes or scales^6,57,58^. Measuring physical activity of animals is not an easy task, but the Rotarod is an acceptably good approximation^59–62^. In fact, we have been able to demonstrate an impairment due to ischemia–reperfusion, which is recovered after an acceptable period of time. This offers another parameter to assess utility of any proposed treatment.

## Conclusions

The system for inducing and maintaining the ischemia designed for this study allows us to reliably simulate the damage associated with the use of a pneumatic cuff in clinical practice, especially in orthopaedic surgery.

The biochemical parameters considered, in particular, CK and LDH levels, and measures of tissue damage (swelling, muscle fibre damage and PMN infiltration) have allowed us to define and characterise the LL-IRI-related damage.

Our results enable us to establish a protocol based on 3 h of ischemia and 3 h of reperfusion as a model of LL-IRI which is representative of local damage, similar to that occurring in clinical practice; but lasting more than usual, is more sensitive, facilitating the detection of differences in future studies of prophylactic treatments against LL-IRI.

## Materials and methods

All the procedures were carried out in accordance with current legislation and were approved by Experimental Animal Ethical Committee of the University of the Basque Country (ref.: M20/2015/075 HERRERO DE LA PARTE). The study was carried out in compliance with the ARRIVE guidelines.

For this study, we used 3- to 4-month-old male WAG/RijHsd rats, with a mean weight of 302 ± 12 g, bred and housed in the animal facilities at the University of the Basque Country (UPV/EHU). Animals were kept in groups of six in type IV cages (ref. 031,200,126, Harvard Apparatus) with wood shavings (F200017, Courbehaye, France) with food (SAFE A04 SP-10, SAFE Complete Care Competence, Augy, France) and water ad libitum, at a constant temperature of 24 °C under a 12-h:12-h light–dark cycle.

### Experimental design

To minimize the influence of circadian variations on the results, all procedures to induce LL-IRI were carried out in the morning. In total, to develop the model and then characterise it, we used 86 animals that were randomised to 11 experimental groups, as shown in Table 2. In all cases, the lower limb subjected to ischemia was the right hind limb.

**Table 2:** Experimental groups. Description of experimental groups and animals used in each group.

Group	Purpose of the group	Number of animals
Pressure	Optimisation of pressure used to induce ischemia	20
Control	Determination of baseline enzyme levels and normal histological structure	6
IR-1	Biochemical analysis after 2 h of ischaemia and 10 min of reperfusion	6
IR-2	Biochemical analysis after 2 h of ischaemia and 1 h of reperfusion	6
IR-3	Biochemical analysis after 2 h of ischaemia and 3 h of reperfusion	6
IR-4	Biochemical analysis after 2 h of ischaemia and 6 h of reperfusion	6
IR-5	Biochemical analysis after 3 h of ischaemia and 10 min of reperfusion	6
IR-6	Biochemical analysis after 3 h of ischaemia and 1 h of reperfusion	6
IR-7	Biochemical analysis after 3 h of ischaemia and 3 h of reperfusion	6
IR-8	Biochemical analysis after 3 h of ischaemia and 6 h of reperfusion	6
IR-9	Histopathological analysis after 3 h of ischaemia and 24 h of reperfusion	6
IR-10	Rotarod (functional) analysis after 3 h of ischaemia and 14 days of reperfusion	6
Total		86

As a first step, we determined the pressure required to produce and maintain ischemia. For this purpose, after exsanguination of the leg with a rubber bandage, the pressure group (PG) animals were subjected to increasingly higher pressures (0.5, 0.6, 0.7, 0.8, 0.9, 1 kg), until the appearance of clinical signs of ischemia (paleness, coldness) and confirmation of a lack of blood flow in the dorsalis pedis artery, with a Doppler laser probe.

Another group of six animals (the control group, CG) was used to determine both the baseline biochemical parameters and the normal histological structure of the gastrocnemius muscle in the lineage of animals used. The pathophysiological response to LL-IRI at the biochemical level was evaluated in ischemia/reperfusion groups 1 to 8 (IR-1 to IR-8), with different durations of ischemia (2 and 3 h) and reperfusion (10, 60, 180 and 360 min). Finally, the ischemia/reperfusion group 9 (IR-9) allowed us to evaluate the consequences at the histopathological level of LL-IRI (after 3 h of ischemia and 24 h of reperfusion).

### System for inducing and maintaining lower limb ischemia

**Figure 5:**
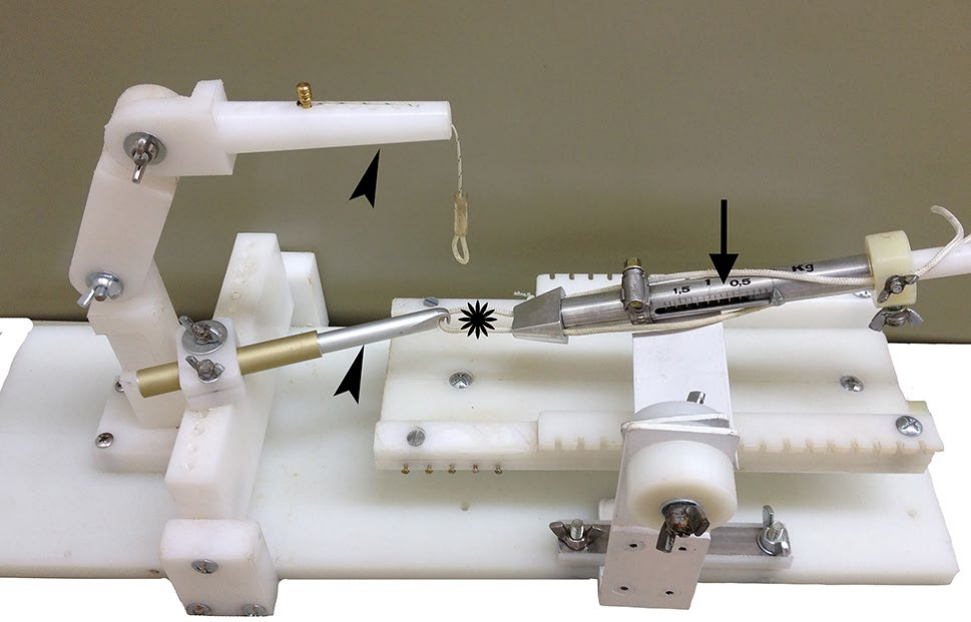
System designed for performing the ischemia. The leg is placed into the loop of the tourniquet (asterisk), adjusting the tensile force with the dynamometer (arrow). The tourniquet is fitted at the top of the thigh with the help of a support arm (lower arrowhead), while the other support arm (upper arrowhead) pulls on the end of the leg.

We used a mechanical system developed in-house to induce and maintain ischemia. It includes an adjustable stand allowing the anaesthetized animal to be placed in a supine position. The tourniquet we used to produce the ischemia was made of a 2-mm diameter nylon cable and this was connected to a piston system and a dynamometer (Fig. 5), allowing adjustment of the pressure applied with the tourniquet to the desired level.

### Anaesthesia and surgical procedure

**Figure 6:**
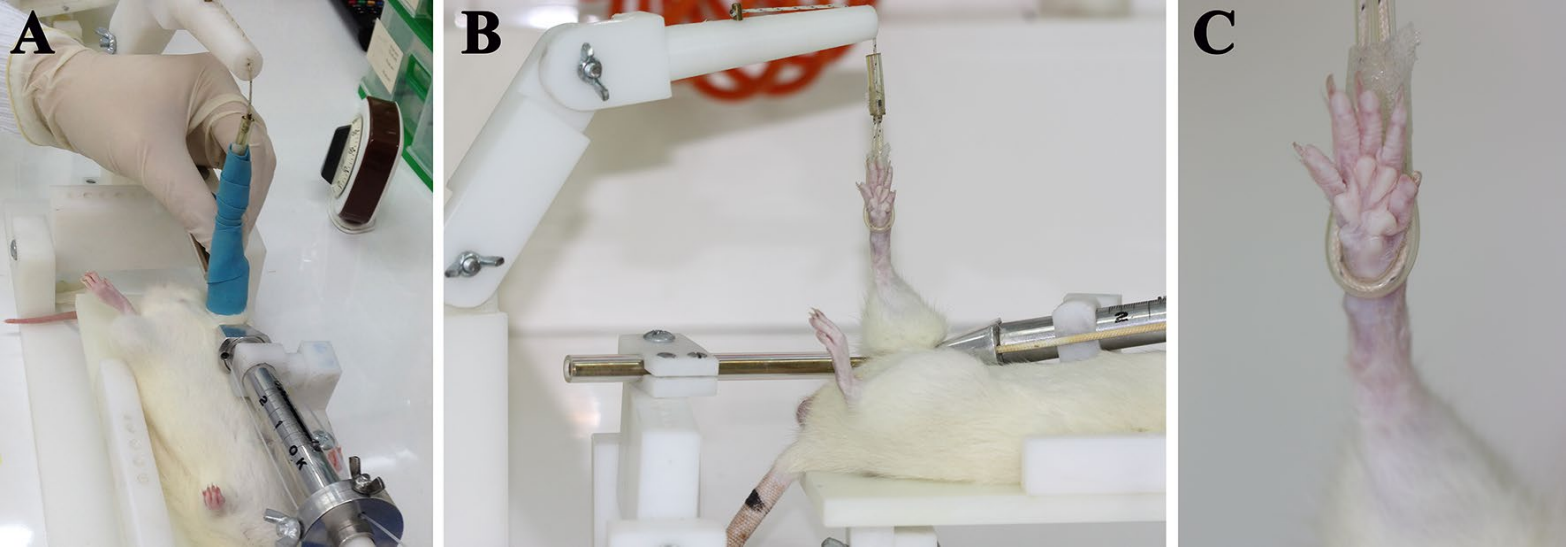
Procedure for inducing ischemia. Exsanguination of the leg is performed with a rubber bandage (**A**), the tourniquet is fitted to the base of the leg and the bandage removed (**B**). It is confirmed that ischemia is maintained by the observation that the leg is pale (**C**).

Animals were anaesthetized using diazepam (15 mg/kg), ketamine (80 mg/kg) and medetomidine (0.5 mg/kg) and placed, supine, on the aforementioned stand. The cable connected to the dynamometer was fixed around the right hind limb, which was held up with a support arm. Once the leg was exsanguinated using a rubber bandage, like an Esmarch bandage (Fig. 6A), the piston was used to tighten the cable to obtain a dynamometer reading of 1 kg and thereby maintain ischemia in the leg (Fig. 6B)^41^. The conditions were confirmed to be truly ischemic by observing a lack of capillary refill and paleness of the leg (Fig. 6C) and using a Doppler laser probe on the area of the dorsalis pedis artery. Halfway through the ischemia, diazepam was administered again (7.5 mg/kg) to keep the animal sedated until the end of the ischemia. After this time (2 or 3 h, depending on the experimental group), we released the pressure applied by the cable, allowing reperfusion in the leg for the period allocated in each experimental group. Restoration of blood flow was confirmed by the presence of clinical signs, in particular, the sudden onset of reactive hyperaemia. For post-ischemia recovery, the animals were placed in cages with wood shavings under a heat lamp at 22–24 °C and checked every 30 min.

### Blood sample collection and biochemical analysis

At the end of the reperfusion period, animals were placed in a supine position under inhalation anaesthesia with isoflurane at 1.5%. We performed wide laparotomy and evisceration to identify the inferior vena cava. Once identified, it was dissected, and using a 21G needle, punctured to collect as high a volume of circulating blood as possible (5–7 ml). These procedures imply the death of the animal, though this was ensured by cervical luxation.

The blood obtained was centrifuged at 3000 rpm for 10 min in II SST™ tubes (BD Vacutainer®, New Jersey, USA). The serum obtained was aliquoted and kept at − 25 °C until analysis.

All the biochemical parameters of interest were measured using a Cobas® 8000 c702 modular analyser (Roche Diagnostics GMBH, Mannheim, Germany). Specifically, we measured following: indicators of kidney function (urea and creatinine levels); levels of electrolytes, namely, sodium (Na +), potassium (K +) and chloride (Cl^−^), ubiquitous enzymes, namely, alkaline phosphatase (ALP), aspartate aminotransferase (AST) and alanine aminotransferase (ALT), and lastly muscle enzymes, namely, creatine kinase (CK) and lactate dehydrogenase (LDH).

### Anatomopathological analysis

Tissue damage was assessed 24 h after the end of the ischemia, by measuring the girth of both legs (experimental and control) and histological analysis of gastrocnemius muscle sections.

Specifically, to assess the swelling caused by LL-IRI, the hind leg girth was measured at the top of the thigh by wrapping a tape measure around the limb, while the animal was anaesthetized with isoflurane at 1.5%. To standardize the procedure, this measurement was taken after shaving the leg (with an electric shaver), and while the animal was on the stand of the ischemia-inducing system with gentle traction applied to the end of the leg using one of the system’s support arms. We measured upper-thigh girth in both experimental (IR) and control (paired control) legs.

After this, animals were sacrificed under anaesthesia by exsanguination through the abdominal aorta. After checking that animals were dead, the gastrocnemius muscles of both legs were removed, weighed and fixed in formaldehyde at 4%. At least 24 h later, specimens were embedded in paraffin, and subsequently, they were sliced into 3-µm histological sections which were stained with haematoxylin/eosin for histological analysis.

Tissue damage was assessed by counting the number of muscle fibres damaged with respect to the total number of fibres observed in five randomly selected fields^36^. We also assessed polymorphonuclear neutrophil (PMN) infiltration, by counting the number of these cells in each field.

### Rotarod test

Six rats were tested on a Rotarod apparatus (Panlab Hardvard Apparatus, Barcelona, Spain). The rats were placed on a moving rod with a rotation speed of 15 rpm. Following the 15 s acclimation time, the Rotarod was gradually accelerated on a 30-s slope, which increased the speed from 15 to 25 rpm.

Once the rod acceleration started, the latency to fall time started to be recorded automatically by the SeDaCom v2.0.03 software (Panlab Hardvard Apparatus, Barcelona, Spain); the speed of the rotarod at the time of fall was also recorded. Prior to ischemia induction, animals were subjected to a 2-day training protocol before being tested on the day -1, which acclimated the rats to the rotarod. The animals were subjected to the Rotarod test every day during the first week after ischemia, and also on day 10 and day 14.

### Statistical analysis

The statistical analysis was carried out using Prism® 7 software (GraphPad Software, San Diego CA, USA). First, the Kolmogorov–Smirnov test was used to check the distribution of the data. Having confirmed that data were normally distributed, the results were expressed as means and standard deviations (SDs), and between-group comparisons were performed with parametric tests.

Comparisons between legs subjected to ischemia/reperfusion and the controls were made using the Student’s t-test for paired samples, while comparisons between the different experimental groups were made using analysis of variance (ANOVA) and the Newman-Keuls post-hoc test. In all the analyses, p values < 0.05 were considered statistically significant.
